# Association Between Familial Hypercholesterolemia and Risk of Cardiovascular Events and Death in Different Cohorts: A Meta-Analysis of 1.1 Million Subjects

**DOI:** 10.3389/fcvm.2022.860196

**Published:** 2022-06-21

**Authors:** Yani Yu, Lei Chen, Honghong Zhang, Zihao Fu, Qi Liu, Haijing Zhao, Yuqi Liu, Yundai Chen

**Affiliations:** ^1^Medical College of Nankai University, Tianjing, China; ^2^Department of Cardiology, The Sixth Medical Center, Chinese PLA General Hospital, Beijing, China

**Keywords:** familial hypercholesterolemia, cardiovascular events, cardiac death, all-cause of death, prognosis, meta-analysis

## Abstract

**Background and Aims:**

The association of familial hypercholesterolemia (FH) with risk of cardiovascular events (CVE) and death in different cohorts is controversial. We aimed to assess the risk of CVE and death in patients with FH in different cohorts, including CHD and ACS patients, White and Asian, different diagnostic criteria.

**Methods:**

We searched PubMed, MEDLINE, and Web of Science electronic databases through May 2021 to identify cohort studies of CVE and death in patients with FH.

**Results:**

We found 18 eligible studies with 1,139,788 participants, including 34,261 patients. There were 31,287 ACS patients, of whom 2,338 were combined with FH. Randomized-effects meta-analysis showed that in patients with FH, relative risk (RR) of CVE and death was 1.87 (95% CI 1.21–2.88), among which CVE was 2.14 (95%CI 1.26–3.64), all-cause of death RR = 1.12 (95% CI 0.89–1.41), and cardiac death RR = 1.03 (95% CI 0.59–1.79). Risk of CVE and death in general population with FH was 2.85 (95% CI 0.72–11.21), hyperlipidemia population RR = 1.59 (95% CI 1.05–2.41), coronary heart disease patients (CHD) RR = 1.46 (95% CI 1.24–1.72), and acute coronary syndrome patients (ACS) RR = 1.71 (95% CI 1.19–2.46). Among ACS patients, the RR of CVE in patients with FH was 1.91 (95% CI 1.55–2.35), the RR of all-cause of death was 1.03 (95% CI 0.80–1.32), and the RR of cardiac death was 1.03 (95% CI 0.59–1.79). The risk of CVE and death in ACS patients with FH in White was 1.69 (95% CI 1.09–2.64) and Asian 1.90 (95% CI 1.31–2.75). RR in patients with Dutch Lipid Network criteria (DLCN) ≥6 vs. <3 points was higher (RR = 2.24, 95% CI 1.69–2.97). RR for long-term follow-up was 1.68 (95% CI 1.09–2.61) and for short-term follow-up was 1.80 (95% CI 1.16–2.78). The results of the overall population were similar, but RR for overall population during a short-term follow-up was 1.49 (95% CI 0.81–2.73). We followed PRISMA checklist to complete meta-analysis.

**Conclusions:**

The risk of CVE and death was increased in patients with CHD, especially in patients with ACS. DLCN ≥ 6 points was suggested for clinical diagnosis of FH. The risk of long-term and short-term CVE and death increased in ACS patients with FH.

**Registration Number:**

INPLASY2021110010.

## Introduction

Familial hypercholesterolemia (FH) is an autosomal dominant hereditary disease characterized by a considerable increase in lifetime total cholesterol (TC) and low-density lipoprotein (LDL), including mutations in *LDLR, APOB, PCSK9* and other genes (1). Exposed to high cholesterol from birth, patients with FH experience the occurrence and development of atherosclerotic lesions in the heart, brain, and peripheral arteries, leading to an increased risk of premature coronary heart disease (CHD), among which acute myocardial infarction (MI) and sudden cardiac death are the leading causes of death (2). Previous systematic reviews estimated that patients with FH had a significantly higher risk of cardiovascular disease (CVD), with or without lipid-lowering therapy (3). A recent meta-analysis of 1.1 million individuals showed that the prevalence rate of FH in the general population is approximately 0.32% (4). Therefore, optimizing lipid management to reduce the incidence of CVD and improve long-term prognosis remains an important clinical and public health issue (5). However, it remains controversial whether there are differences in the different cohorts of cardiovascular events among patients with FH, especially those with CHD or ACS. In addition, the clinical diagnostic criteria for FH have been controversial. Although gene diagnosis is the gold standard of FH diagnosis, gene diagnosis is not suitable for large-scale population screening because of its high cost. So the purpose of this study was to explore the effects of FH on cardiovascular outcomes in different cohorts through meta-analysis in short-term and long-term follow-up, and to explore the effectiveness of clinical diagnostic criteria relative to gene diagnosis in FH patients.

## Methods

### Search Strategy

We did a meta-analysis about the association between Familial Hypercholesterolemia and risk of cardiovascular events (CVE) and death in different cohorts according to PRISMA guidelines. We searched the PubMed, MEDLINE, and Web of Science databases to identify cohort studies reporting the outcome of CVE and death in patients with FH. The article was published from database inception to June 2021. We also reviewed the reference lists of relevant articles to identify additional studies. A broad search strategy based on MeSH Term was used, as follows: (“familial hypercholesterolemia” OR “familial hypercholesterolaemia” OR “familial hypercholesterolemic” OR “familial hypercholesterolaemic” OR “hyperlipoproteinemia type II”) AND (“prognosis” OR “follow-up”). We use this extensive search strategy to avoid missing potential studies. Two researchers independently screened the literature, evaluated quality, and extracted data.

### Inclusion and Exclusion Criteria

Inclusion criteria were: (1) cohort study; (2) included participant groups with and without FH; (3) outcome was at least one cardiovascular event (including non-fatal MI, angina, percutaneous coronary intervention or coronary artery bypass grafting, heart failure, stroke, TIA and peripheral vascular disease) or death (all-cause death or cardiac death); (4) provided risk ratio, survival curve, or event rate to calculate the relative risk ratio (RR); and (5) If the same study publishes results from different periods, include the most recent study data.

Exclusion criteria were: (1) duplicate or missing information; (2) meta-analysis or systematic review; (3) only included children; (4) conference summaries, guidelines, case reports, letters, and similar reports; (5) animal studies, randomized controlled trials, or diagnostic tests; and (6) We a-priori excluded studies with non-English language owing to quality concerns.

### Data Extraction and Quality Assessment

Two investigators independently extracted data, including the first author's name, date of publication, country, cohort years, sample size, number of events, follow-up time, and hazard ratio (HR) or RR. In the case of disagreement, a third researcher was consulted. Exposure data included the definitions and criteria for FH, number of participants, and duration of follow-up. Outcome data included the definitions of cardiovascular outcomes, number of participants with and without FH, multivariate-adjusted risk estimates (RR, HR, or odds ratio [OR]), and variables included in the multivariate analysis. Because the included studies are cohort studies, We used the Newcastle–Ottawa Scale (NOS) to evaluate the quality of the included studies. Two researchers evaluated independently. If the opinions are inconsistent, it shall be solved by a third party.

### Statistical Analysis

Stata 16.0 software was used for meta-analysis (StataCorp LLC, College Station, TX, USA). The RR and 95% confidence interval (CI) were used together with the effect size. The results of each cohort study were reported as RR, HR, OR, or binary frequency data. We used algebraic methods to convert the OR and frequency data to RR. If feasible, we used adjusted risk estimates from a multivariate model (6).

We performed a separate meta-analysis using the DerSimonian–Laird random-effects model to obtain a pooled RR for each outcome measure and the primary endpoint of CVE and death. When multiple outcomes were reported, we analyzed the results of cardiovascular events, cardiac death, and all cause death.

We used Cochran's Q test to assess differences between studies, and the *I*^2^ statistic was used to quantify the proportion of inconsistencies observed in the results. Values of *I*^2^≥ 50% and *P* ≤ 0.10 indicated no heterogeneity among studies and a fixed-effect model was used for analysis. We also used Cochran's Q test to calculate the heterogeneity between subgroups (7). Sensitivity analysis was performed for the results of the meta-analysis, and a funnel plot was drawn for publication bias analysis. If there was publication bias, we used the trim-and-fill method and Egger's test to verify whether publication bias affected the stability of the combined effect size. The test level was α = 0.05. Subgroup analysis was performed based on outcome events, study population, diagnostic criteria, follow-up time, age, and weather adjust risk factors.

## Results

### Study Selection and Characteristics

**Figure 1:**
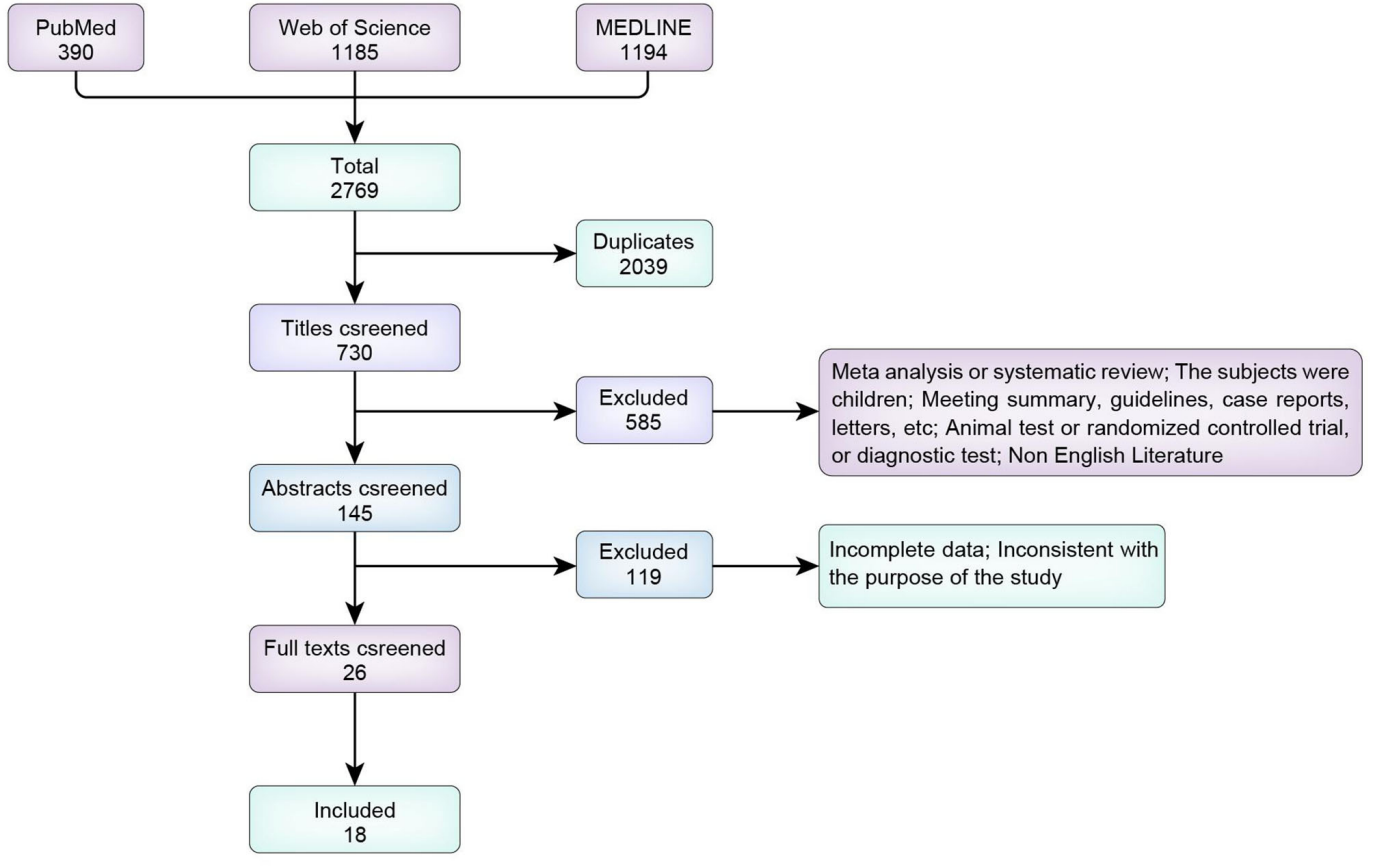
Flow chart of study selection.

We screened a total of 2,769 articles identified in PubMed, MEDLINE, and Web of Science. We excluded 2,039 duplicate articles, including meta-analyses and reviews (*n* = 64); animal studies (*n* = 11); studies including children (*n* = 140); meetings, guidelines and case reports (*n* = 68); reports with missing information (*n* = 29); diagnostic experiments and treatment plans (*n* = 265); and non-English articles (*n* = 9). Initially, 145 studies were included. We excluded 119 irrelevant articles after reading the abstract and 8 after reading the full text; thus, 18 articles were finally included (Figure 1).

Table 1 summarizes the characteristics of the included studies, conducted from 2001 to 2020. The study population included the overall population, participants with hyperlipidemia, CHD and ACS. Diagnostic criteria included clinical and genetic criteria, most of which were in accordance with the Dutch Lipid Clinical Network (DLCN) criteria: LDL level, physical examination (xanthoma of tendon and corneal arch), early-onset CHD, and family history. Some studies considered FH to be present with a DLCN score ≥3 and non-FH was defined as DLCN <3 points; other studies used a score ≥6 to define FH. According to the clinical Simon Broome (SB) criteria, TC level >290 mg/dl or LDL >190 mg/dl combined with an early family history of premature CHD indicates a possible FH, and combined with signs of hypercholesterolemia (presence of chordoma tendinea in patients or family members), this indicates a definite FH. Some studies used the Modified Make Early Diagnosis To Prevent Early Death (MEDPED) criteria, as follows: TC ≥ 270 mg/dl and LDL ≥ 200 mg/dl for patients 19–29 years old; TC ≥ 320 mg/dl and LDL ≥ 220 mg/dl for patients 30–39 years old; TC > 340 mg/dl and LDL > 240 mg/dl for patients age 40 years or older. Other diagnostic criteria included the 2017 Japan Atherosclerosis Society (JAS) guidelines for adults, as follows: high LDL cholesterolemia (untreated LDL ≥180 mg/dl); tendon xanthoma (including 9 mm hypertrophy of Achilles tendon) or nodular xanthoma; a family history of FH or early-onset coronary artery disease in first- or second-degree relatives; adults (older than age 15 years) who met two or more of the above criteria could be second-degree relatives, and two or more of the above in an adult over 15 years of age could be diagnosed as FH. American Heart Association (AHA) criteria were as follow: LDL >190 mg/dl and family history of premature CHD in a first-degree relative. Other less commonly used clinical criteria were used in some studies. Eleven studies were conducted in patients with ACS.

**Table 1:** Characteristics of the included studies.

**References**	**Year**	**Area**	**Population characteristics**	**FH criteria**	**Sample size**, ***n***		**Age**		**Lipid lowering therapy**		**Previous CV events (MI)**		**Follow-up, years**	**Outcomes**	**Events**	**Adjusted risk**
					**FH**	**Non FH**	**FH**	**Non FH**	**FH**	**Non FH**	**FH**	**Non FH**				
Yasuda et al. (8)	2001	Japan	Youth AMI	Clinical other	13	23	35 ± 5	36 ± 5	Undefined	Undefined	undefined	undefined	9.4	Cardiogenic death + recurrent of myocardial infarction + angina pectoris	24	
Takasaki et al. (9)	2020	Japan	ACS	Clinical other	18	688	55.5 ± 12.22	70 ± 12.95	94.4%	90.4%	5.65%	6.7%	1	MACE	70	
Khan et al. (10)	2020	United Kingdom	HLP	gene	87	170	48.9 ± 14.9	56.7 ± 10.6	62%	51%	17%	9%	11	MACE	72	
Rerup (11)	2016	Denmark	AMI	DLCN≥3	1281	11893	59.1 ± 11.48	65.7 ± 13.63	93.15%	88.3%	37%	19%	3.3	all-cause of death + recurrence of myocardial infarction	4466	Yes
Rallidis et al. (12)	2016	Greece	Youth STEMI	DLCN≥6	65	255	32.7 ± 2.6	32.4 ± 3.5	97.2%	73.9%	0%	0%	9.2	MACE	99	Yes
Silva et al. (13)	2016	Brazil	HLP	gene	515	303	42.3 ± 16.8	50.9 ± 15.1	72.6%	53.5%	11.98%	3.03%	1	MACE	47	
Jung et al. (14)	2018	Korea	Common	Modified MEDPED	1257	501709	45.8 ± 13	44.3 ± 11.16	0%	0%	undefined	undefined	14.6	all-cause of death	23413	Yes
Iyen et al. (15)	2019	United Kingdom	Common	SB / DLCN≥8	14097	42506	42.5 ± 11.7	41.6 ± 12.5	75.54%	20.14%	0%	0%	13.8	MACE	6202	Yes
Dyrbus et al. (16)	2019	Poland	ACS & Stable CHD	DLCN≥3	3074	16707	59.2 ± 10.4	65.6 ± 9.9	91.7%	86.7%	49.37%	30.90%	5	MACE	4845	
Singh et al. (17)	2019	United States	Youth MI	DLCN≥6	180	1816	45.5 ± 5.56	45 ± 4.44	89.4%	90%	0%	0%	11.2	all-cause of death	228	Yes
Tscharre et al. (18)	2018	Austria	ACS & Stable CHD	DLCN≥6 VS <3	77	1141	56 ± 11.2	66.3 ± 11.7	96.1%	92.1%	27.3%	20.7%	6	MACE	437	Yes
Danchin et al. (19)	2020	France	AMI	DLCN≥6VS分<3	146	5001	53 ± 12	65 ± 14	99%	90%	16%	14%	5	all-cause of death + AMI + stroke	1339	Yes
Masana et al. (20)	2019	Spain	Common	Clinical other	12823	514177	61.7 ± 13.4	50.2 ± 20	88.9%	6.7%	undefined	undefined	5	ASCVD	30555	Yes
Nanchen et al. (21)	2016	Switzerland	ACS	DLCN≥6	73	4461	49.8 ± 9.3	63.4 ± 12.3	94.5%	96.7%	undefined	undefined	1	MACE	275	Yes
Svendsen et al. (22)	2021	Norway	AMI	gene	211	1947	38.5 ± 15.9	38.1 ± 15.7	undefined	undefined	0%	0%	17	all-cause of death + recurrence of myocardial infarction	899	Yes
Al-Rasadi et al. (23)	2017	Arabian Gulf	ACS	DLCN≥6 VS <3	111	2034	50 ± 10	63 ± 12	98%	96%	undefined	undefined	1	ASCVD	300	Yes
Auckle et al. (24)	2017	China	STEMI	DLCN≥3	203	243	50.1 ± 3.9	53.5 ± 4.2	97.7%	99.2%	undefined	undefined	1	MACE	108	
Wang et al. (25)	2019	China	Youth CHD	DLCN≥6 VS <3	30	453	31 ± 2.22	33 ± 2.96	75.7%	81.7%	undefined	undefined	5	MACCE	82	Yes

Fourteen studies assessed cardiovascular events, including non-fatal MI, angina, percutaneous coronary intervention or coronary artery bypass grafting, heart failure, stroke, TIA and peripheral vascular disease. Seven studies assessed all-cause of death, and four studies assessed cardiac death. The mean age of the 18 studies was 47.86 ± 16.24 years, 52.20 ± 15.39 years in the FH group and 47.73 ± 16.25 years in the non-FH group. However, baseline data for modified MEDPED were not provided in Keum Ji Jung's research (14). Therefore, the data in Table 1 are MEDPED standards.

### Quality Evaluation

The NOS scale was used to evaluate the quality of the 18 cohort studies (Table 2). The results of evaluation showed that all studies had scores ≥5, indicating high quality. These 18 studies were included in the meta-analysis. Some used logistic regression to predict the incidence of events in the population lost to follow-up. Half of the studies considered the time factor, and the effect index was HR.

**Table 2:** Methodological quality evaluation of the included studies.

**References**	**Exposure group representativeness**	**Non-exposure group representativeness**	**Ascertainment of exposure**	**Demonstration that outcome of interest was not present at start of study**	**Comparability**	**Blinding**	**Follow up long enough for outcome**	**Adequacy of follow up of cohorts**	**Quality scores**
Yasuda et al. (8)	⋇	⋇	⋇	⋇	~	~	⋇	⋇	6
Takasaki et al. (9)	⋇	⋇	⋇	⋇	~	⋇	⋇	⋇	7
Khan et al. (10)	⋇	⋇	⋇	~	~	⋇	⋇	⋇	6
Rerup et al. (11)	⋇	⋇	⋇	⋇	⋇⋇	⋇	⋇	⋇	9
Rallidis et al. (12)	⋇	⋇	⋇	⋇	⋇	⋇	⋇	~	7
Silva et al. (13)	⋇	⋇	⋇	⋇	~	⋇	~	⋇	6
Jung et al. (14)	⋇	⋇	⋇	⋇	⋇	⋇	⋇	⋇	8
Iyen et al. (15)	⋇	⋇	⋇	⋇	⋇	⋇	⋇	⋇	8
Dyrbus et al. (16)	⋇	⋇	⋇	⋇	~	⋇	⋇	~	6
Singh et al. (17)	⋇	⋇	⋇	⋇	⋇	⋇	⋇	⋇	8
Tscharre et al. (18)	⋇	⋇	⋇	⋇	⋇⋇	⋇	⋇	⋇	9
Danchin et al. (19)	⋇	⋇	⋇	⋇	⋇	⋇	⋇	⋇	8
Masana et al. (20)	⋇	⋇	⋇	⋇	⋇	⋇	⋇	⋇	8
Nanchen et al. (21)	⋇	⋇	⋇	⋇	⋇⋇	⋇	⋇	⋇	9
Svendsen et al. (22)	⋇	~	⋇	⋇	⋇	⋇	⋇	⋇	7
Al-Rasadi et al. (23)	⋇	⋇	⋇	⋇	⋇	~	⋇	⋇	7
Auckle et al. (24)	⋇	⋇	⋇	⋇	~	⋇	~	⋇	6
Wang et al. (25)	⋇	⋇	⋇	⋇	⋇	⋇	⋇	⋇	8

### Overall Risk of Cardiovascular Events and Death

**Figure 2:**
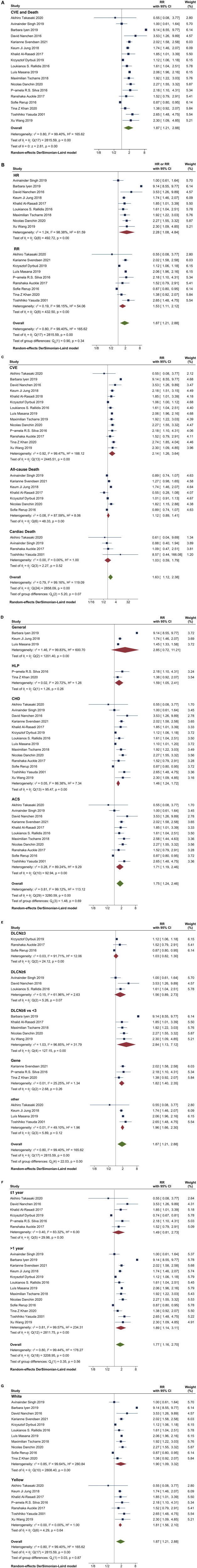
Comparison of the incidence of prognostic events. **(A)** the overall pooled RRs of CVE and death in FH patients; **(B)** Adjusted HR and non-adjusted RR of CVE and death in FH patients. **(C)** RR of CVE, all-cause death and cardiac death; **(D)** RR of CVE and death in different cohorts, including general population, Hyperlipidemia population, CHD and ACS population; **(E)** RR of different diagnostic criteria; **(F)** RR of short-time and long-time follow-up; **(G)** RR of Whites and Asians.

The heterogeneity among studies was significant (*I*^2^= 99.4%, *P* < 0.001), so we used the random-effects model for meta-analysis. Different subgroup analyses of patients with or without FH are shown in Supplementary Table 1. The overall pooled RR for incident CVE and death among participants with FH was 1.87 (95% CI 1.21–2.88) (Figure 2A). When separately analyzing studies with HR and binomial frequency as the effect size, the results showed that the risk of CVE and death in patients with FH was increased, after adjusting for time factors (HR = 2.28, 95% CI 1.08–4.84); the combined RR of non-adjusted time factors was 1.53 (95% CI 1.11–2.12) (Figure 2B). RR for CVE was 2.14 (95% CI 1.26–3.64), all cause of death 1.12 (95% CI 0.89–1.41), cardiac death 1.03 (95% CI 0.59–1.79), and overall RR 1.63 (95% CI 1.12–2.38) (Figure 2C).

**Figure 3:**
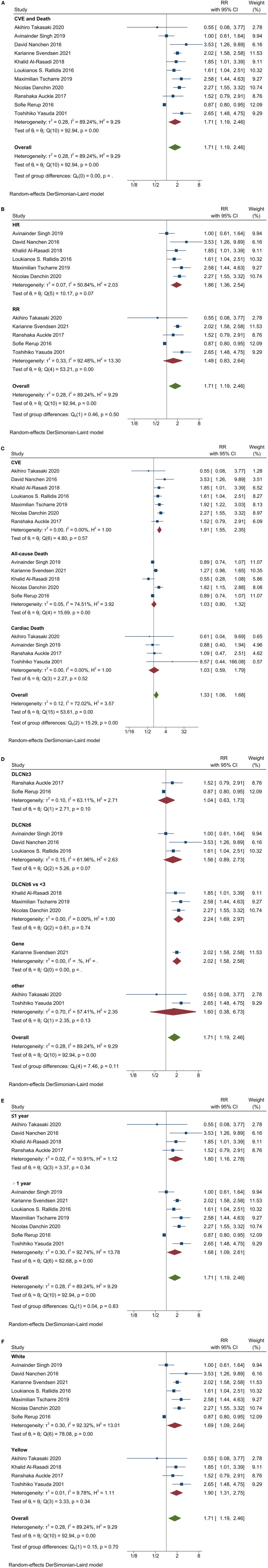
Forest plots analysis performed in different cohorts of ACS patients with FH. **(A)** the overall pooled RRs of CVE and death; **(B)** Adjusted HR and non-adjusted RR of CVE and death. **(C)** RR of CVE, all-cause of death and cardiac death; **(D)** RR of different diagnostic criteria; **(E)** RR of short-time and long-time follow-up; **(F)** RR of Whites and Asians.

The overall pooled RR of CVE and death in ACS patients with FH was 1.71 (95% CI 1.19–2.46) (Figure 3A). Similarly, the risk of ACS with FH increased after adjustment for time (HR = 1.86, 95% CI 1.36–2.54); the RR of non-adjusted time factors was 1.48 (95% CI 0.83–2.64) (Figure 3B). RR of CVE was 1.91 (95% CI 1.55–2.35), all cause of death 1.03 (95% CI 0.80–1.32), cardiac death 1.03 (95% CI 0.59–1.79), and overall RR 1.33 (95% CI 1.06–1.68) (Figure 3C).

### Cardiovascular Events and Death in Different Cohorts

We conducted subgroup analysis for different cohorts. The RR for general population, hyperlipidemia, CHD, ACS and overall were 2.85 (95% CI 0.72–11.21), 1.59 (95% CI 1.05–2.41), 1.46 (95% CI 1.24–1.72), 1.71 (95% CI 1.19–2.46) and 1.75 (95% CI 1.24–2.46), respectively (Figure 2D). The RR showed no significant increase in the whole population.

Subgroup analysis was performed in ACS patients. Using DLCN as the standard score, the score ≥6 was FH, and the scores <3 was non-FH, RR was 2.24 (95%CI 1.69–2.97) (Figure 3D). The results were similar in the overall population, with RR 2.84 (95%CI 1.13-7.12) and 1.82 (95%CI 1.40–2.35) for three studies using genetic criteria (Figure 2E).

Patients with ACS and FH had an increased risk in follow-up ≤1 year and >1 year (RR = 1.80, 95%CI 1.16–2.78; RR = 1.68, 95% CI 1.09–2.61) (Figure 3E). However, in the overall population, patients with FH had no significant increase in the RR of CVE and death at ≤ 1 year of follow-up (RR = 1.49, 95% CI 0.81–2.73) (Figure 2F).

The risk of CVE and death in patients with FH was no related to ethnicity, regardless of the ACS patients or the overall population. The RR of ACS patients with FH was 1.69 (95% CI 1.09–2.64) in white patients and 1.90 (95% CI 1.31–2.75) in Asian patients (Figure 3F). While in overall population, the RR was 1.90 (95% CI 1.09–3.32) in white patients and 1.81 (95% CI 1.56–2.10) in Asian patients (Figure 2G).

### Heterogeneity Testing

There was significant heterogeneity between studies (*I*^2^ = 99.3%, Supplementary Table 1). Among ACS patients, there was little heterogeneity in studies of cardiac death and CVE (*I*^2^= 0%); There is still considerable heterogeneity in studies of all-cause of death (*I*^2^= 74.51%). Therefore, different prognostic events were considered to be important sources of heterogeneity (*P* = 0.00) (Figure 3C). Similar results were found in the overall population (*P* = 0.07, Figure 2C), and we believe that different diagnostic criteria are also an important source of heterogeneity in the overall population (*P* = 0.00) (Figure 2F). Although Cochran's Q-test showed small heterogeneity between subgroups of different study populations, follow-up time, race, and whether or not there were risk factors (*P* > 0.1), studies grouped by the above factors showed large differences in RR (Figures 2D,F,G; Supplementary Figure 1A). We did a subgroup analysis by age (Supplementary Figure 1B). The subgroup analysis of FH age larger than non-FH was not significantly different. On the contrary, the results of FH smaller than non-FH group were significantly different. Therefore, we believe that age differences do not fully explain the heterogeneity of this study.

### Sensitivity Analysis

We performed a sensitivity analysis to test the robustness and sources of heterogeneity of our meta-analysis results. First, we used the method of omitting one study at a time to conduct a sensitivity analysis for risk of total cardiovascular events and death (Supplementary Figure 2; Table 3). Elimination of any one of the 18 studies did not have a significant impact on the total RR. Second, we combined the studies with the outcome variables RR and HR, respectively, because HR contains time variables, which is an important factor causing the difference of results. Our results showed that the combined RR and HR were different from the overall results, but the results were not statistically significant (Figures 2A–C). In sum, we considered the results of this meta-analysis is relatively stable.

**Table 3:** Sensitivity analysis of the included studies.

**References**	**Estimate**	**95%CI**	
Takasaki et al. (9)	0.6541	0.2121	1.096
Singh et al. (17)	0.6566	0.2073	1.1059
Iyen et al. (15)	0.5039	0.2725	0.7353
Nanchen et al. (21)	0.5882	0.142	1.0345
Svendsen et al. (22)	0.6136	0.1593	1.0679
Jung et al. (14)	0.6228	0.164	1.0816
Al-Rasadi et al. (23)	0.6193	0.1703	1.0683
Dyrbus et al. (16)	0.6498	0.1645	1.1351
Rallidis et al. (12)	0.6325	0.1833	1.0817
Masana et al. (20)	0.6062	0.0399	1.1725
Tscharre et al. (18)	0.6169	0.1667	1.0672
Danchin et al. (19)	0.6065	0.1554	1.0576
Silva et al. (13)	0.6101	0.1616	1.0586
Auckle et al. (24)	0.6307	0.182	1.0794
Rerup et al. (11)	0.6682	0.2137	1.1226
Khan et al. (10)	0.6375	0.187	1.088
Yasuda et al. (8)	0.5981	0.1489	1.0472
Wang et al. (25)	0.6073	0.1592	1.0554
Combined	0.6191	0.1834	1.0548

### Publication Bias

Supplementary Figure 3 presents a funnel plot for evaluating publication bias in the included studies. The funnel plot did not show an obvious symmetrical inverted funnel. The RR value obtained by interpolating the right side of the funnel plot was 2.418 (95% CI:1.668–3.505); the results of an Egger's regression test were P>| Z |=0.6254.

## Discussion

In this meta-analysis, we found that the latest current evidence from 18 longitudinal studies involving 1,139,788 participants showed that the risk of CVE and death was significantly increased in patients with FH, and the RR was higher after adjusting for time factors, (Supplementary Figure 1A). Similar results were found in ACS subjects. The data show that clinical criteria and genetic diagnostic criteria (gold standard) have a similar ability to predict the prognosis risk while DLCN≥6 points (diagnosis of definite or probable FH) and DLCN <3 points (diagnosis of unlikely FH) had the highest risk prediction ability. Therefore, greater attention is needed toward patients with a DLCN score ≥6 owing to a higher clinical prognosis risk.

One of the most important problems with FH at present is its low detection rate. However, the detection rate of FH in patients with CHD, especially ACS, is 10 times higher than that of the general population (4, 26). Our study showed that patients with FH have a higher risk of CVE and death in high-risk populations, including CHD and ACS, while there is no significant difference in CVE and death risk among subjects without CHD (Supplementary Figure 1C). Therefore, FH screening may have greater clinical value in the implementation of secondary prevention of CHD. Perez et al. (27) found increased age, male, history of ASCVD, hypertension, body mass index, active smoking, and elevated LDL-C and Lp(a) levels as independent prospective predictors of increased risk of ASCVD in patients with FH.

Owing to the imperfect medical record system in most hospitals and incomplete information needed for a clinical diagnosis of FH (including physical examination such as tendon xanthoma or corneal arch), especially in retrospective studies, the rate of missed diagnosis of FH is high. Gene testing is the gold standard for FH diagnosis. The genetic diagnostic subsets in this study were highly homogenous. However, due to its high cost, it is difficult to be widely used in clinical practice. The number of subjects with genetic diagnosis in this study was only 2.37% (*n* = 3,233), which was a defect of the study. Nanchen et al. found that the prevalence of FH detected using three different clinical diagnostic criteria differed, and the prognosis risk also differed (21). We think all patients with early-onset CVD or severe hyperlipidemia should be examined for corneal arch and tendon xanthoma to improve the diagnostic rate of FH; these may be a simpler clinical diagnostic standard. Additionally, the clinical diagnostic criteria for patients with ACS have not been put into practice. Some studies state that the DLCN diagnostic criteria are not fully applicable for patients with ACS. and the prevalence of clinical diagnoses of FH ranges from 1 to 14% in different study cohorts of ACS (17). Genetic testing should be performed in young patients with ACS and patients with high LDL cholesterol level to identify patients with FH and relatives at high risk in a timely manner (28).

There was no significant difference in cardiac death, and all-cause of death between patients with and without FH, except for CVE. This may be owing to the lack of comprehensive evaluation of outcome events because MACE events showed significant differences between these groups. Mundal et al. obtained similar results, in that there was no significant difference in the standardized mortality ratio (SMR) of FH all-cause of death, and the SMR of cardiac death was significantly higher (29). Some study showed that in addition to CHD, the risk of stroke/TIA/peripheral vascular disease was also significantly increased (15, 30), while other suggest that the risk of fatal stroke in FH diagnosed using the SB criteria was not significantly increased (31). Takasaki et al. found that the recurrence rate of cardiovascular events in patients with ACS and FH was not higher than the rate in patients without FH in short-term follow-up (9). There was no significant difference in the risk among patients who had MI with or without FH in a Chinese population, without adjusting for risk factors (24). This may be related to underestimation of the FH prevalence owing to a decrease in LDL levels 12 to 24 h after the occurrence of ACS or the overestimation of FH prevalence owing to the lack of consideration of polygenic hypercholesterolemia in clinical diagnosis (32–34). In our study, the longer the follow-up, the worse the prognosis, which is consistent with the long-term process of FH induced CVE and death. However, ACS patients with FH had a significantly increased risk of CVE and death at < 1 year of follow-up, which was inconsistent with the overall results. There were significant differences in short-term follow-up outcomes among patients with FH, which may be related to differences in follow-up duration and whether only patients with acute myocardial infarction were included and the use of different medications cannot be ruled out. More trials should be conducted in the future to assess the outcomes of FH patients, especially ACS patients. Secondly, the difference of LDL-C compliance rate is also one of the reasons. The proportion of subjects receiving lipid-lowering treatment in FH group was significantly higher than that in non FH subjects. Due to cascade screening or selective survival, some FH patients received early treatment and improved lifestyle, which means that susceptible individuals die early, so the risk of survival is relatively low (35). Although most studies did not provide dose information of lipid-lowering treatment, the treatment of FH was seriously insufficient, and high-intensity statins and PCSK9 inhibitors were not better applied to FH patients. Finally, different FH definition standards will certainly lead to the deviation of the results. Dyrbuś et al. found that the mortality rate in patients with FH at 1 month was significantly higher than that in patients without FH; however, at 1-year, 3-year, and 5-year follow-up, no significant difference was observed between the two groups. After propensity score matching, patients with FH had significantly higher all-cause of death during 3-year and 5-year follow-up (16). On the contrary, Rerup et al. found that patients with MI and FH had a higher risk of recurrent MI but there was no significant difference in all-cause of death (11), which was associated with the use of DLCN >3 to diagnose FH (36).

In this study, the current data results do not indicate a significant difference in the risk of prognosis of FH between Whites and Asians, which may be related to the different populations and diagnostic criteria. And the number and proportion of cases involving Asians are very small. Some Asian studies have shown that the DLCN and SB criteria are not applicable to Asian populations, with some changes having been proposed (37, 38). For example, Jung et al. found that the improved MEDPED standard is most consistent with the FH phenotype among Korean populations (14).

## Conclusion

Our results showed that patients with FH had a higher RR of CVE and death. The more co-morbidities and longer follow-up, the higher RR of CVE, but ACS patients with short-term follow-up also had a higher risk of CVE and death. young FH patients may have a greater risk of prognosis. These findings suggest that clear clinical diagnostic criteria (such as DLCN score ≥6 vs. <3) or genetic testing should be used to diagnose FH so as to reduce missed diagnoses of FH and accurately identify patients with FH and prognostic risk, for timely assessment of whether high-intensity lipid-lowering therapy is needed to improve poor clinical prognosis. (Registration Number: INPLASY2021110010. There is no modification to the protocol or registration information).

## Limitations

First, the heterogeneity among studies was significant, which may be owing to the different populations, diagnostic criteria, follow-up times, and defined outcome events. Different diagnostic criteria and outcome events in particular contribute more to the heterogeneity among studies. Second, some studies did not report the rate of loss to follow-up, so we could not accurately evaluate bias owing to loss to follow-up nor whether this bias is an important source of heterogeneity and whether it could lead to overestimation of the prognosis risk of FH. Third, our subgroup analysis was a non-paired analysis; we cannot exclude the influence of the differences in factors other than grouping factors (i.e., confounding factors) on subgroup comparisons. However, this is a common shortcoming in subgroup analyses of all systematic reviews. Finally, the number of FH subjects was significantly less than that of non FH subjects, which is an irresistible factor and may lead to overestimation of the results. Therefore, we need simple and accurate screening methods to improve the diagnosis rate of FH.

## Data Availability Statement

The original contributions presented in the study are included in the article/Supplementary Material, further inquiries can be directed to the corresponding authors.

## Author Contributions

YL and YC designed the research. YY and LC performed the experiments and analyzed the data. YY wrote original draft. LC, HZang, and ZF collected the data and revised the article. QL and HZhao revised and prepared the figures. All authors reviewed and agreed to the manuscript.

## Funding

This work was supported in part by Beijing Lisheng Cardiovascular Health Foundation Pilot Fund key projects to YC, the National Natural Science Foundation of China under Grant 81827808, and the Chinese Cardiovascular Health Alliance -Advanced Fund under Grant 2019-CCA-ACCESS-054.

## Conflict of Interest

The authors declare that the research was conducted in the absence of any commercial or financial relationships that could be construed as a potential conflict of interest.

## Publisher's Note

All claims expressed in this article are solely those of the authors and do not necessarily represent those of their affiliated organizations, or those of the publisher, the editors and the reviewers. Any product that may be evaluated in this article, or claim that may be made by its manufacturer, is not guaranteed or endorsed by the publisher.

## Supplementary Material

Supplementary Figure 1 Forest plots analyzed by different cohorts. **(A)** The RR for whether to adjust the risk factors; **(B)** RR for age comparison between FH group and non FH group; **(C)** The RR for whether it is CAD.

Supplementary Figure 2 Sensitivity analysis for risk of total cardiovascular events and death.

Supplementary Figure 3 Publication bias graph.

Supplementary Table 1 Analysis of different subgroups with or without FH.
