# Supplementary material for: Association Between Familial Hypercholesterolemia and Risk of Cardiovascular Events and Death in Different Cohorts: A Meta-Analysis of 1.1 Million Subjects
Source: Front Cardiovasc Med. 2022 Jun 21;9:860196. doi: 10.3389/fcvm.2022.860196 (PMC9253470; doi:10.3389/fcvm.2022.860196)
Supplement: Supplementary Figure 1 — Forest plots analyzed by different cohorts. (A) The RR for whether to adjust the risk factors; (B) RR for age comparison between FH group and non FH group; (C) The RR for whether it is CAD. [file Data_Sheet_1.zip › Supplementary Figure2-3 Table1.pdf]

Supplement Figure 2: Sensitivity analysis for risk of total cardiovascular events and death.

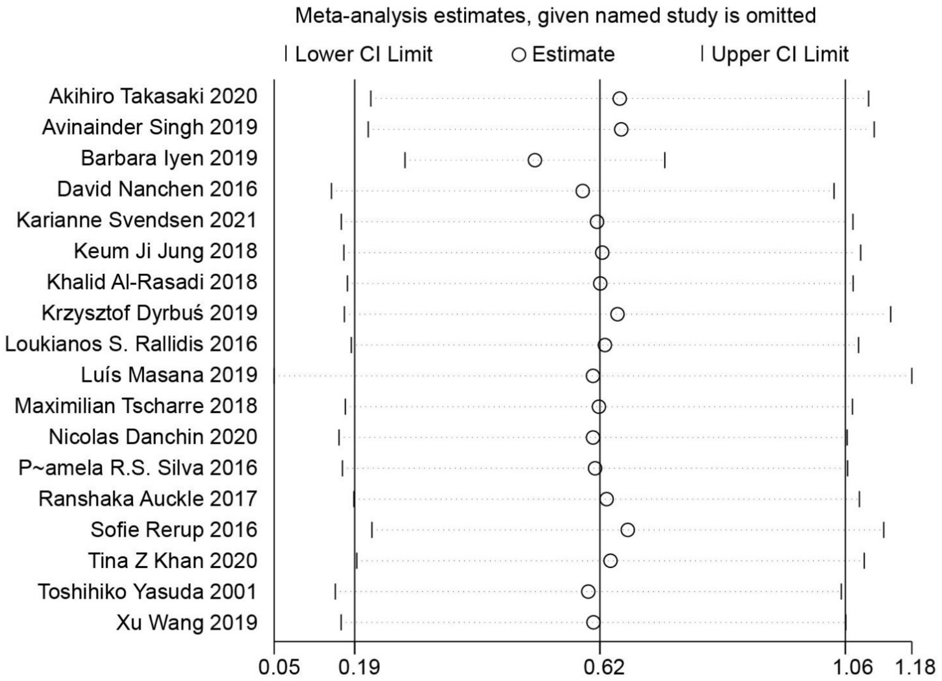

Supplement Figure 3: Publication bias graph.

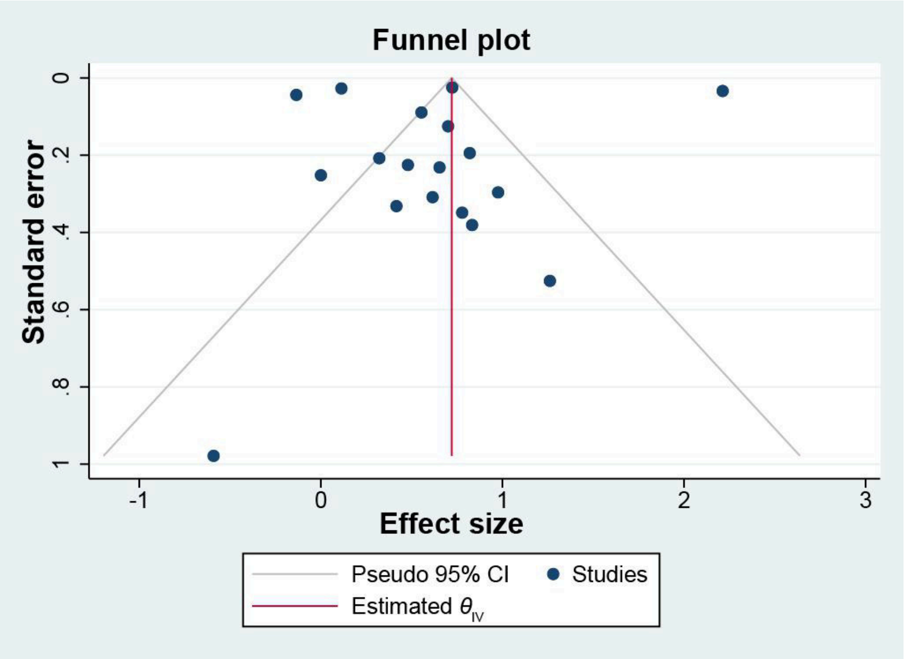

Supplement table 1. Analysis of different subgroups with or without FH

Subgroup	Number	Effect Model	RR（95%CI）	Heterogeneity test	
				I^2%	P
**Outcome**					
Cardiovascular events	18	random	1.87（1.21-2.88）	99%	＜0.0001
all-cause of death	7	random	1.12（0.89-1.41）	86%	＜0.0001
Cardiac death	4	fixed	1.03（0.59-1.79）	0%	0.52
**Study Population**					
Whole population	3	random	2.85（0.72-11.21）	100%	＜0.0001
Hyperlipidemia population	2	fixed	1.59（1.05-2.41）	21%	0.26
Coronary heart disease population	14	random	1.46（1.24-1.72）	86%	＜0.0001
ACS population	11	random	1.71（1.19-2.46）	89%	＜0.0001
**Diagnostic Criteria**					
clinical diagnosis					
Dlcn ≥ 3 points	3	random	1.03（0.82-1.30）	92%	＜0.0001
Dlcn ≥ 6 points	3	random	1.56（0.89-2.73）	59%	0.09
Dlcn ≥ 6 vs < 3	5	random	2.84（1.13-7.12）	97%	＜0.0001
Other clinical diagnosis	4	fixed	1.96（1.66-2.30）	49%	0.12
gene diagnosis	3	fixed	1.82（1.40-2.35）	25%	0.26
**Race**					
white person	11	random	1.90（1.09-3.32）	99.64%	＜0.0001
yellow race	7	fixed	1.81（1.56-2.10）	0%	0.64
**Follow up time**					
Short term (≤ 1 year)	6	random	1.49（0.81-2.73）	83%	＜0.0001
Long term (> 1 year)	13	random	1.89（1.14-3.11）	100%	＜0.0001
