# Supplementary figures and images for: Association Between Familial Hypercholesterolemia and Risk of Cardiovascular Events and Death in Different Cohorts: A Meta-Analysis of 1.1 Million Subjects
Source: Front Cardiovasc Med. 2022 Jun 21;9:860196. doi: 10.3389/fcvm.2022.860196 (PMC9253470; doi:10.3389/fcvm.2022.860196)

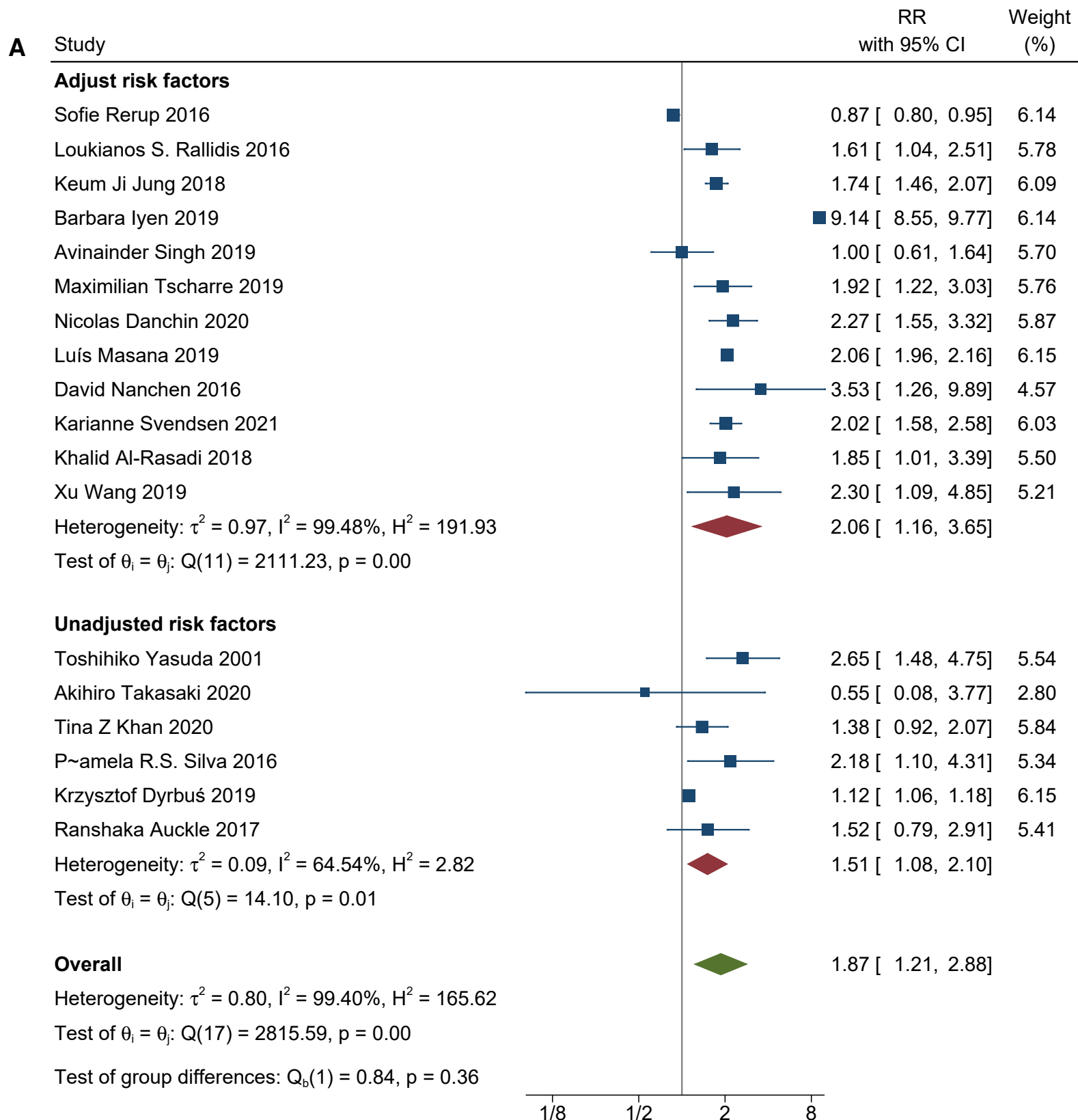

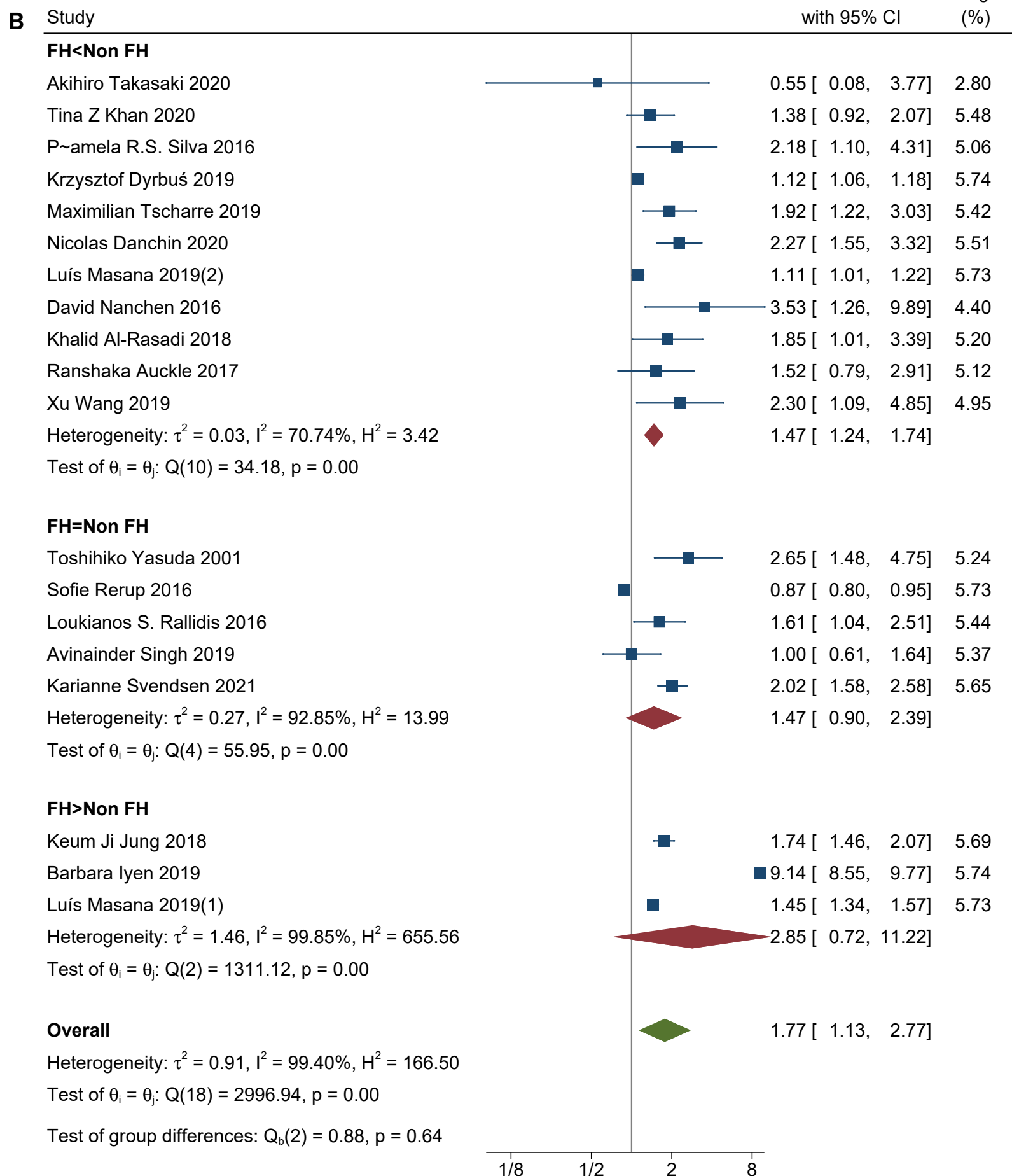

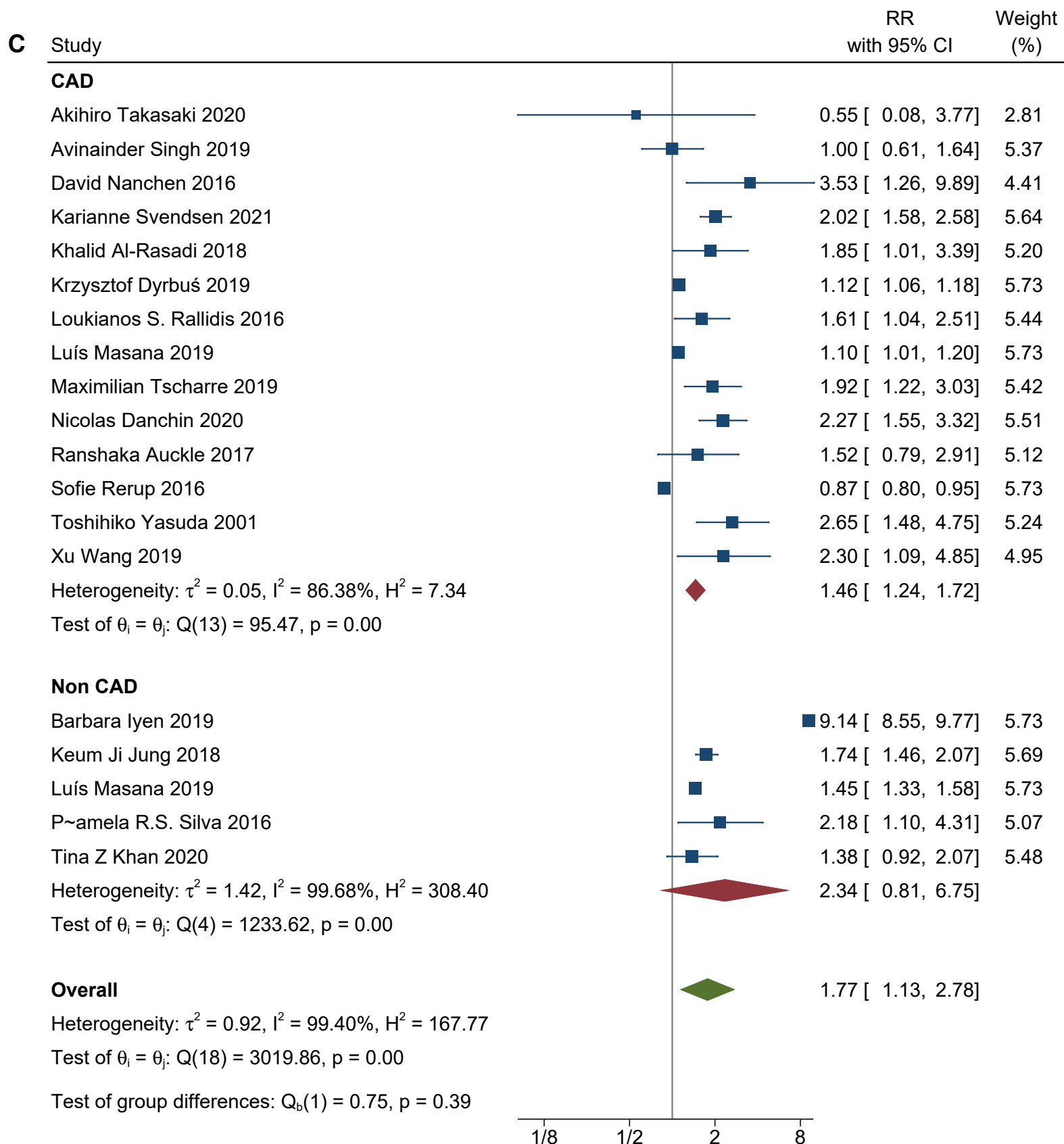

Supplement: Supplementary Figure 1 — Forest plots analyzed by different cohorts. (A) The RR for whether to adjust the risk factors; (B) RR for age comparison between FH group and non FH group; (C) The RR for whether it is CAD. [file Data_Sheet_1.zip › Supplementary Figure1.pdf]
